# Selective killing of malignant B cells using T cells redirected against malignancy variant receptor

**DOI:** 10.1186/2051-1426-2-S3-P16

**Published:** 2014-11-06

**Authors:** Chungyong Han, Sujung Sim, Kwang Hui Kim, Don Gil Lee, Ho Sik Oh, Sang Hyun Park, Sunhee Hwang, Won Young Kim, Sangeun Lee, Young Ho Kim, Beom Kyu Choi, Carl June, Byoung Se Kwon

**Affiliations:** 1 National Cancer Center, Gyeonggi-do, Republic Of Korea; 2 University of Pennsylvania, Philadelphia, PA, USA

## Background

Advances in gene-transfer system and in-depth understanding of immune mechanism have made the immunotherapy a powerful tool for fighting against cancers. Recent studies demonstrated a therapeutic potential of T cells with chimeric antigen receptor (CAR) targeting CD19 in refractory hematopoietic malignancies. At the same time, however, hence the CD19 targeting results in normal cell destruction such as B cell aplasia, a novel marker that specifically expressed in malignant B cells should be applied. In this study, we developed anti-malignancy variant receptor (MVR) mAb that exclusively bound to malignant B cells but not to normal B cells, and demonstrated that autologous T cells expressing CAR construct with anti-MVR scFv (MVR-CAR T cells) efficiently suppressed the outgrowth of malignant B cells in lymphoid organs.

## Results

Malignant B cell-specific monoclonal antibody was isolated from the Balb/c mice immunized with Burkitt's lymphoma cell line, L3055. The antibody specifically recognized the established B lymphoma cell lines and malignant B cells derived from acute lymphoblastic leukemia, chronic lymphocytic leukemia, and diffuse large B cell lymphoma patients. Q-TOF analysis revealed that anti-MVR mAb recognized one of the CD74 variants that distinctively expressed in malignant B cells. We used anti-MVR mAb to generate CAR T cells for the rapid and efficient production of autologous T cells targeting malignant B cells. MVR-CAR T cells were generated by stimulating T cells with anti-CD2, CD3, CD28 Ab-coated beads and transducing MVR-CAR construct using lentiviral vector system. Autologous MVR-CAR T cells efficiently induced cytotoxicity against EBV-transformed LCLs but not against the normal CD19^+ ^B cells *in vitro*. Furthermore, when the MVR-CAR T cells were adoptively transferred into immune-deficient RAG2^-/-^γc^-/- ^mice into which LCLs were subcutaneously injected 3 weeks previously, they efficiently suppressed the outgrowth of metastasized LCLs in secondary lymphoid organs *in vivo*.

## Conclusions

We developed anti-MVR mAb - a novel malignant B cell-specific antibody. Anti-MVR mAb recognized one of CD74 variants that exclusively expressed on malignant B cells. MVR-CAR T cells successfully induced LCL-specific cytotoxicity *in vitro *and *in vivo*. Considering the unique specificity on malignant B cells, anti-MVR mAb can be a therapeutic of B cell malignancies without normal B cell destruction.

